# Traumatic Spinal Injury in Children; Time to Revise Pre-Hospital and Diagnostic Protocols?

**DOI:** 10.3390/jcm13082372

**Published:** 2024-04-18

**Authors:** Michelle Oude Alink, Huub Stassen, Jochem Spoor, Jeroen Renkens, Xavier Moors, Marjolein Dremmen, Robert Jan Stolker, Caroline van der Marel

**Affiliations:** 1 Department of Anesthesiology, Erasmus University Medical Center, 3015 GD Rotterdam, The Netherlands; h.stassen@erasmusmc.nl (H.S.); x.moors@erasmusmc.nl (X.M.); r.stolker@erasmusmc.nl (R.J.S.); c.vandermarel@erasmusmc.nl (C.v.d.M.); 2 Department of Neurosurgery, Erasmus University Medical Center, 3015 GD Rotterdam, The Netherlands; j.spoor@erasmusmc.nl; 3 Department of Orthopedic Surgery and Sports Medicine, Erasmus University Medical Center, 3015 GD Rotterdam, The Netherlands; j.renkens@erasmusmc.nl; 4 Helicopter Emergency Medical Services, Erasmus University Medical Center, 3015 GD Rotterdam, The Netherlands; 5 Department of Radiology and Nuclear Medicine, Erasmus University Medical Center Rotterdam, 3015 GD Rotterdam, The Netherlands; m.dremmen@erasmusmc.nl

**Keywords:** traumatic spinal injury, pediatric, trauma, imaging, MRI, CT

## Abstract

**Background**: Traumatic spinal injury in children is a rare but serious life event. Predicting pediatric patients at risk for spinal injury remains difficult. This study focuses on the cause of the injury and predictors to identify children at risk and appropriate diagnostic procedures. **Methods**: Retrospective chart review from the Landelijke Trauma Registratie of patients with spinal injury from 2010 to 2021 in a level 1 pediatric trauma center. **Results**: We included 114 children with spinal injury, 79.8% of whom were aged 12–17 years. In the overall trauma population, the incidence of spinal injury was 10% in children aged 12–17 years, 2.3% in children aged 6–11 years, and 0.4% in children 0–5 years of age. Neurological deficits were present in 27.2% of patients in the emergency department, with permanent deficits in 14.0%. Spinal fractures were present in 91.2% of 12–17-year-olds, 43.8% in 6–11-year-olds, and 71.4% in 0–5-year-olds. ISS was 23 (SD 14) in children with spinal injury compared to 8 (SD 9) for children without spinal injury. **Conclusions**: In children 0–11 years old, spinal injury is very rare compared to the overall trauma population, and there are more non-osseous injuries. Clinicians should consider MRI as the next step after conventional X-ray to diagnose or exclude spinal injuries in this group. In older children aged 12–17 years, the incidence of spinal injury is much higher, at 10%. Although ISS is higher in children with spinal injury, a low ISS does not exclude spinal injury. If one fracture is found, more fractures in other regions of the spine may be present.

## 1. Introduction

Traumatic spinal injury can be a serious life event and is a major cause of disability and mortality. Highly specialized and demanding care is needed for these patients across the globe. The Global Burden of Disease Study in 2019 estimates 0.9 million incident cases, 20.6 million prevalent cases, and 6.2 million years lived with a disability of complete spinal cord injury in adults and children globally [1,2]. The outcomes after medical treatment are highly variable and strongly dependent on the level and severity of injury. In adults, many studies have been conducted to assess the etiology and prognosis of traumatic spinal injury. The American Spinal Injury Association (ASIA) Impairment Scale is commonly used for prognosis and monitoring.

Pediatric spinal cord injury is relatively uncommon, contributing to 3.0% of all traumatic spinal cord injuries in developed countries and 4.5% in developing countries, but it can have a large impact on quality of life and can bring a heavy burden of disease [3,4].

Cervical spinal injuries show an incidence of 0.5–1.85% in the overall pediatric trauma population [5,6]. Despite this low relative incidence, further exploring the relation between trauma mechanism, age, and level of injury is necessary to be able to further optimize prevention of secondary damage, diagnostics, and treatment.

At three years of age, the anterior and posterior arches of the atlas are fused, ossifying at seven years of age [7,8]. The odontoid process fuses with the axis at six years of age, eventually completely ossifying around the age of 12 [9,10]. The entirety of the C2 is ossified in the third decade [9]. The spine below the axis generally has mature vertebral bodies and fused anterior transverse processes at the age of 6 [9]. At the age of 25 the spinous processes and epiphyseal discs are fused to the vertebrae and the development of the spine can be seen as complete [7].

Due to active growth and these differing compositions of bone and cartilage in the different age groups, combined with changes in activities during childhood and bodily proportions, we hypothesize that the characteristics of spinal column injury in children varies with age and varies more than in adults.

In the pre-hospital situation in many countries, spinal boards, cervical collars, and/or vacuum mattresses are used to immobilize patients with possible spinal column injuries. In the Netherlands in 2016, spinal boards and cervical collars for children were abandoned by the ambulance services due to lack of evidence and potential harmful side effects such as pressure ulcers [11].

Currently, it remains challenging to predict based on clinical findings which pediatric trauma patient suffers from clinically significant spinal injury. A recent Cochrane review on pediatric cervical spine injuries shows that prediction models used in the adult population guiding diagnostic strategies do not seem accurate in the pediatric population, especially for younger children [5]. The updated PEDSPINE II can help to identify patients at risk but is focused on the cervical spine only [12].

Internationally conventional X-ray and computed tomography scan (CT scan) are commonly used by hospitals as the first choice to assess spinal column injury in trauma patients. Considering the increased risk of leukemia and brain cancer after ionizing radiation for CT scanning in early life, a possible the next step could be magnetic resonance imaging (MRI) after conventional X-ray to reduce the long-term risk of cancer and accurately diagnose or rule out spinal injuries [12,13,14]. Furthermore, MRI is able to visualize non-osseus tissues, contrary to CT scanning [15,16,17]. The new Dutch guidelines, implemented in 2022, incorporate these risks for the pediatric population and advise conventional X-ray as the first step, with dedicated MRI reserved for patients with neurological deficits [14].

With this retrospective cohort study, we aim to find the incidence of spinal trauma in the overall trauma population and the differences between age groups. Furthermore, we want to use this to find further parameters to determine best strategies for diagnostic procedures for pediatric trauma patients.

## 2. Materials and Methods

### 2.1. Background

In the Netherlands, we have a system of prehospital selection of patients by the emergency medical service (EMS) and helicopter emergency service (HEMS) to determine the appropriate hospital for each patient. Patients with minor injuries are transferred to the nearest hospital. Patients with (suspected) severe injuries or specific characteristics such as children or possible intracranial injuries are directly transported by EMS/HEMS to a hospital with the required expertise.

The Erasmus Medical Center is one of the eight level 1 pediatric neurotrauma centers in the Netherlands. It services the crowded urban area of Rotterdam, as well as the surrounding rural areas, with approximately 2.2 million inhabitants [18]. For spinal or neurotrauma patients in particular, the adherence area is larger, as the Erasmus Medical Center also functions as a referral center for the surrounding hospitals in case a patient requires care that cannot be provided in the other hospital.

The Landelijke Trauma Register (National Trauma Registry), or “LTR,” is a database where all trauma patients in the Netherlands in all emergency departments (ED) are registered. This database entails patients with traumatic injury coded according to the AIS injury-coding system within 48 h after the event in the Netherlands [18,19]. These patients must have been admitted (to a generic ward or intensive or medium care unit), been transferred to a different hospital, or died at the ED (excluding death on arrival) [18]. All patients had to score an Injury Severity Score (ISS) of 2 or higher [18]. Since the start of this database in 2007, approximately 70,000–80,000 patients a year are registered nationally, with 12% 0–9 years of age and 7% 10–19 years of age [18]. This gives a total of approximately 14,000 children a year with a registered trauma.

### 2.2. Study Design and Data Collection

We conducted a retrospective cohort study, according to the strobe criteria for retrospective studies, on pediatric patients in the LTR of the Erasmus Medical Center in Rotterdam. Completed data in the LTR were available from 2010 to 2021.

Detailed data were obtained from all pediatric patients 0–17 years of age at the time of injury, with at least one coding in the LTR indicating spinal injury. As the ethical approval did not entail all trauma patients, we obtained pre-analyzed data from the LTR on the total number of trauma patients per age group, gender, trauma mechanism, and mortality.

Data collected from the LTR included age, gender, trauma mechanism, type of trauma, HEMS dispatch, hospital admittance, ICU admittance, level of injury, traumatic brain injury, ISS, and mortality.

All spinal injury patients were cross-checked with hospital records on exclusion criteria and to obtain additional data on injuries, treatment, morbidity, and mortality.

Information regarding spinal CT scan in the ED was available from 2017 to 2021, and only spinal injury patients from this period were used for this section, as well as for cause of trauma, which was not registered before 2013 in the LTR.

To assist with analysis and provide answers for the aim of our study, patients were divided into different age categories based on fusion of the vertebrae at the age of 6 and the change from elementary to high school. The age categories were 0–5 years, 6–11 years, and 12–17 years.

### 2.3. Inclusion and Exclusion Criteria

Patients 0–17 years of age with a registered injury to the vertebral column or spinal cord documented in the LTR were included. Spinal injuries had to be confirmed by neurological exam or radiographical diagnosis. 

Patients with congenital deformity of the spinal column or injury due to a non-traumatic event (for example, pathological fractures) were excluded.

### 2.4. Data Analysis

For analysis, we used IBM SPSS Statistics version 28.0.1.0 (Armonk, NY, USA: IBM Corp.). Descriptive statistics were used for most analysis due to the small and heterogenic groups. The one-way ANOVA was used for differences in means between age groups.

Missing data are described per variable in Section 3.

### 2.5. Ethics Approval

The Medical Ethical Committee of the Erasmus University Medical Center Rotterdam (MEC-2022-0491) approved this study.

All data were anonymously stored in a protected database for analysis. To protect patients’ privacy, we did not include details that could be traced back to individual patients.

## 3. Results

### 3.1. Inclusion and Exclusion

Within the 12-year period, 125 patients met the inclusion criteria. The exclusion criteria were met by 11 patients after cross-referencing the hospital records. Due to severe congenital deformity of the spinal column, two patients were excluded, as they did not suffer from a clinically significant trauma. Nine patients were excluded because their clinical signs were diagnosed as psychiatric after thorough clinical examination and imaging. Finally, 114 patients were analyzed.

### 3.2. Demographics, ISS, and Total Trauma Population

**Figure 1:**
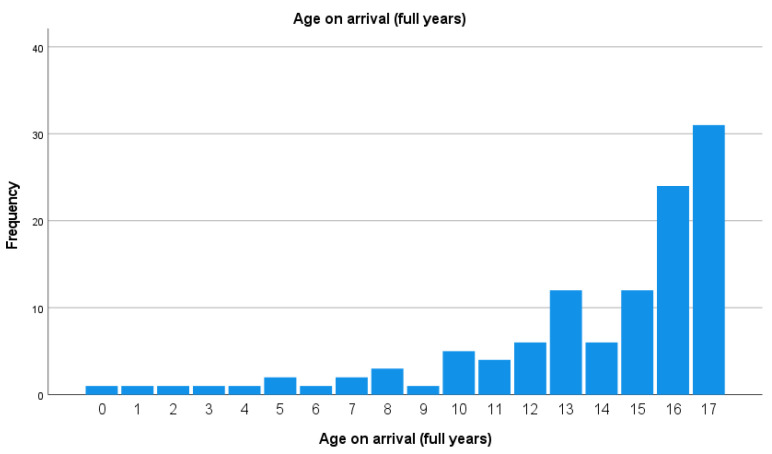
Age distribution of children with spinal trauma.

The group characteristics are shown in Table 1. The majority of patients were aged 12–17 years (79.8%), with a mean of 14.3 years. The group of 0–5-year-olds consisted of only seven children. The age distribution is shown in Figure 1.

Males represented 57.9% of the overall spinal trauma group.

The Injury Severity Score (ISS) showed a mean score of 23 (range 14), with no significant difference between age groups (*p* = 0.450 at 95% CI). This is higher than the overall trauma group, which showed a mean ISS of 8 (Table 1).

Traumatic spinal injury represented 7.1% of the overall pediatric trauma group. In the group of 0–5-year-olds this was 0.4%; in 6–11-year-olds it was 2.3%, and in 12–17-year-olds it was 10.0% (Table 1).

### 3.3. Cause of Injury

Cause of trauma was registered in the LTR from 2013; this was registered for 98 patients.

The majority of patients (42 (42.9%)) suffered from a traffic-related accident, followed by accidents at home in 26 patients (26.5%) (Table 2).

Attempted suicide was the cause of injury in 27 trauma patients, while 14 (51.9%) had a spinal column injury. All were aged 12–17 years, and 11 (78.6%) were female (Table 2).

### 3.4. Level of Injury and Associated Injuries

Injuries were divided by their anatomical regions—cervical, thoracic, lumbar, and sacral—for further analysis.

**Figure 2:**
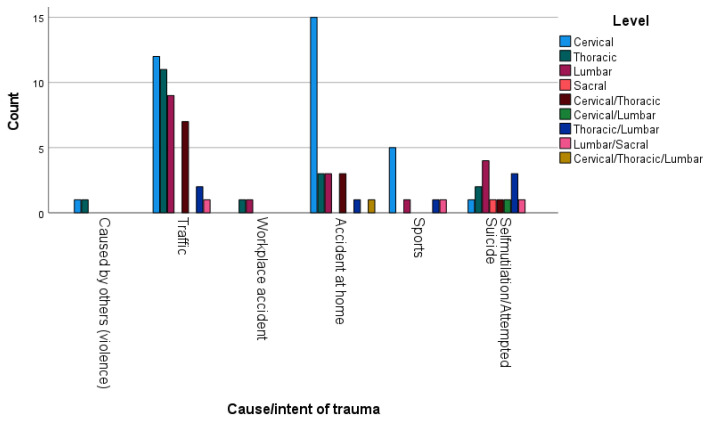
Cause of trauma and level of injury.

**Figure 3:**
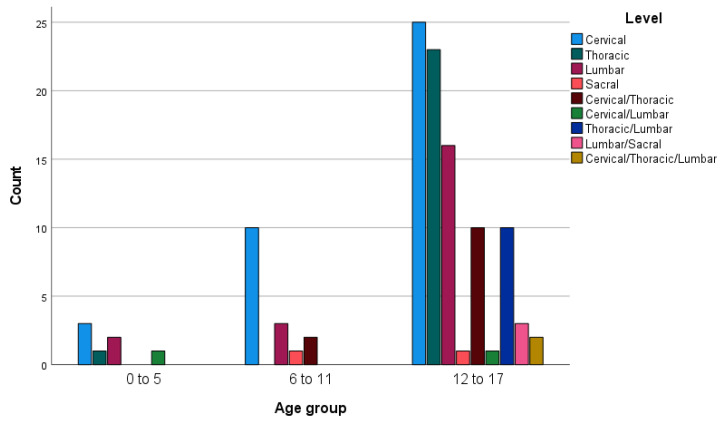
Age distribution and level of injury.

In 45 patients (39.5%), there was a single vertebra involved. For 35 (30.7%) patients, there were multiple vertebrae involved, and in 29 subjects (25.4%), vertebrae in multiple regions were involved (Table 3, Figure 2 and Figure 3).

**Figure 4:**
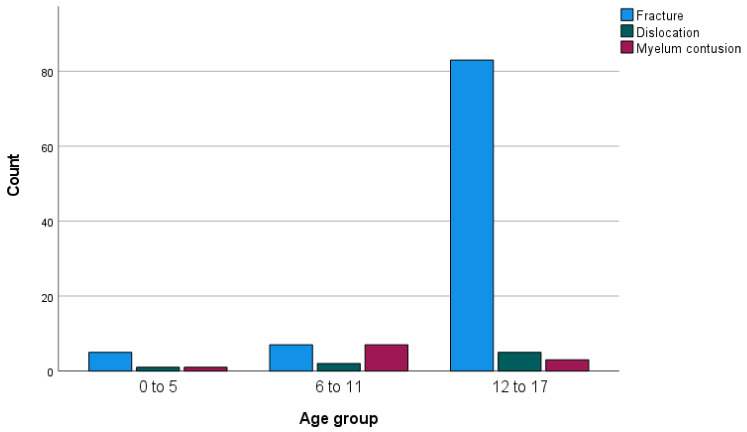
Pathology per age group.

In five patients, contusion of the spinal cord without osseous injuries was found, with four on the cervical level and one on the thoracic level. Three contusions occurred in the group of 6–11-year-olds, one in the group of 0–5-year-olds, and one in the group of 12–17-year-olds. Fractures were most prevalent in the group of 12–17-year-olds, where 91.2% of patients had a fracture. The incidence was 71.4% in the group of 0–5-year-olds and 43.8% in the group of 6–11-year-olds (Figure 4).

After accidents at home, most patients suffered from isolated cervical injuries. Of these 16 patients, the injuries of 6 were due to privately owned playground equipment—mostly trampolines. Six were due to falls from the stairs or open windows, and four others were due to various trauma mechanisms.

In this cohort, associated traumatic brain injury was present in 52 (45.6%) patients. In patients with injuries in the cervical region this number was 23 (46.0%), in the thoracic region it was 23 (48.9%), in the lumbar region it was 13 (34.2%), and in the sacral region it was 1 (20.0%) (Table 3).

Figure 3 shows the age distribution of the level of injuries. In the relatively small age group of 6–11-year-olds, isolated cervical spinal column injury was more common, whereas in the older age group of 12–17-year-olds, more combined injuries were found.

### 3.5. Neurological Deficits

To determine neurological deficits, we only analyzed the survivors due to the fact that the patients who died during their hospital stay had lethal traumatic brain injury; therefore, clinical neurological deficit due to spinal column injury could not be determined.

Due to the heterogenic character, neurological deficits are described in more detail in Supplementary Materials Addendum A. Neurological deficit was found in 27.2% of the children. These 26 patients are described in detail in Supplementary Materials Addendum A, 10 of whom (38.5%) recovered completely. However, in 16 patients (14.0%), the neurological deficits were permanent. 

The age range of the patients with neurological deficits was 5–17 years.

Partial spinal cord injury was diagnosed in 14 (53.8%) patients. Of this group, five made a full recovery, eight recovered partially, and one showed no recovery. Surgical intervention for spinal trauma was indicated in 10 patients, 2 of whom received a HALO-vest, 1 of whom received a cervical collar, and 1 of whom did not require any intervention.

In seven (26.9%) patients, there was a complete spinal cord injury, one of whom suffered a severe central cord lesion due to contusion without osseous injury. All these patients received surgical intervention. In four patients, there was no clinical recovery, one made a partial clinical recovery, and one made a partial clinical recovery at one level but no recovery of complete spinal cord injury at a lower level. The patient with the contusion made a full recovery. The injuries were on the cervical, thoracic, and lumbar levels.

Five patients (19.2%) showed sensory symptoms only, four made a full recovery, and one recovered partially. Two of these patients needed surgical intervention.

One of the 26 patients in Supplementary Materials Addendum A had clinical suspicion of neurological spinal cord deficit. Due to the severity of the traumatic brain injury, the extent and recovery could not be determined. Although this patient survived, the exact neurological follow-up concerning the neurologic deficits due to the spinal injury is lacking.

Two patients suffered from progressive neurological symptoms, one of whom reported to the ED 24 h after the trauma due to the progression of symptoms. Both patients were immobilized according to the older ambulance protocol with a cervical collar and spinal board, and both received urgent surgical decompression due to the progression of symptoms. One made a full recovery after surgery, and the other had a neurological recovery of the upper extremities but not of the lower extremities.

### 3.6. Diagnostics

During the period 2017–2021, a total of 402 pediatric trauma patients underwent a CT of the spine. Of these 258 patients 12–17 years of age, 73 were 6–11 years of age and 70 were 0–5 years of age. Spinal injury was diagnosed in 52 (12.9%) patients during this period. Of these, 42 (16%) were aged 12–17 years, eight (11%) patients were aged 6–11 years, and two (2.8%) patients were aged 0–5 years.

### 3.7. Treatment

Surgical intervention such as repositioning, fixation, and/or decompression was required for 24 children (21.1%). Five (5.5%) patients were treated with a HALO vest, and 17 (14.9%) needed non-surgical immobilization by, for example, a neck collar. 

The other 68 (59.6%) patients did not receive intervention for their spinal column injury. In five of these patients, intervention was not considered due to the severity of the intracranial injuries.

Neurological deficits were present in 15 (71.4%) patients who received operative treatment, and in 6 patients (40.0%) this improved after intervention.

### 3.8. Mortality

In this cohort, 10 patients were deceased, of whom 9 died within 30 days of the trauma. All these patients had severe neurological trauma, with a GCS ranging from 3 to 5 on arrival by EMS/HEMS, which was also the main cause of death in each patient. Two patients were hemodynamically instable due massive hemorrhage. Five out of ten patients required cardiopulmonary resuscitation before arriving at the ED, all of which was initially successful. 

No patient died due to their spinal injury.

## 4. Discussion

In the overall cohort of pediatric trauma patients in our hospital, the incidence of spinal injury was 10.0% in children aged 12–17 years. This is much higher than we anticipated. Previous studies showed an incidence of 0.5–1.85% of cervical spinal injuries in pediatric trauma patients [5,14]. The high incidence in our cohort might be selection bias due to pre-hospital selection by emergency medical services (EMS) and helicopter emergency medical services (HEMS), which transfer the more critically injured children to a level 1 pediatric trauma center. Furthermore, we included all spinal injuries, not only those in the cervical region. Further studies in the overall national trauma population are necessary to determine whether this is representative for the entire trauma population aged 12–17 years.

Most injuries to the spinal column occurred in the 12–17-year-old age group, with 79.8%, which is in line with earlier research regarding this topic [1,3,12]. The incidence of spinal column injury in this cohort drastically increases around the age of 10.

In the younger age groups, the incidence of spinal trauma was very low, with 0.4% in 0–5-year-olds and 2.3% in 6–11-year-olds.

As previous studies show, it is difficult to predict pediatric patients at risk for (cervical) spinal injury [5]. Our study is unfortunately no exception to this. Although the ISS was higher in the group with spinal injury, we found a large range, so a low ISS does not exclude spinal injury. The same is true for high- or low-velocity impact.

Associated traumatic brain injury was present in 45.6% of patients. This may be used as an indicator but not as a proper predictor. The traumatic brain injury was also a main cause of mortality besides major bleeding in all patients.

In this cohort, all patients with neurological deficits experienced them directly after trauma and were diagnosed by the EMS/HEMS. The two patients with progressive neurological deficits only presented to the ED after this deterioration. However, several patients required surgical interventions for spinal injuries without neurological deficits.

Possibly due to the fear of missing a clinically significant spinal injury, many CT scans of the spine were performed in this trauma population. In the youngest group of 0–5-year-olds, only 2.8% of the patients who underwent spinal CT suffered a spinal injury. This suggests that during the study period, we were using too much ionizing radiation, especially in this youngest group of children. In the older groups, there were relatively more positive findings on CT, with 11% in 6–11-year-olds and 16% in 12–17-year-olds.

The CHILDSPINE study also shows that in previous years, not only MRI but also CT scanning in the ED has increased, despite the risks of developing leukemia or brain cancer with ionized radiation [12,13].

In the Netherlands, future studies will have to show whether the new diagnostic protocol that was implemented in 2022 has reduced the rate of CT scans for these patients [14].

The fact that 4 of the 23 children ≤12 years of age suffered from spinal cord injury without osseous injury emphasizes the need to choose imaging techniques that are capable of detecting these injuries in young children.

As also stated by the authors of the CHILDSPINE study, careful consideration should be given to determine whether further imaging is required after conventional X-ray [12,14]. Although MRI is more and more available in the emergency setting in many hospitals, not all hospitals will have the capacity to use it for all pediatric trauma patients.

Future prospective studies are required to determine in which patients a “wait and see” policy is appropriate. Until then, clinicians in the ED should carefully consider which diagnostic strategy is best for their patients.

For a patient with suspected life-threatening injuries, a CT will remain the first choice after conventional X-ray and ultrasound to determine further treatment.

However, for stable pediatric trauma patients, conventional X-ray and neurological examination should be the first step. In the case of clinical suspicion of spinal injury, an MRI should be considered as the next step, especially in cooperative children.

Young or distressed children might need anesthesia for an MRI, which may not be needed for CT scanning. Previous studies did not show negative affects concerning anesthesia for MRI in small numbers of pediatric trauma patients [12,14,15,20]. However, the APRICOT trial showed that, especially in children, there are risks concerning anesthesia [21]. Therefore, the risks of anesthesia and the chance of spinal trauma need to be weighed in each case.

Traffic accidents were the type of trauma that contributed the most to spinal column injury in children, followed by accidents at home. This is similar to other studies showing traffic accidents, sports, and accidents at home as the most likely causes [4,6,22].

Traumatic spinal column injury across all ages is most prevalent in males, with the incidence rates being about twice as high as the incidence rates for females [2,4,6,22]. Our study is no exception, with 57.9% of patients being male.

Two trauma mechanisms stood out in this cohort. First were isolated cervical spine injuries from accidents at home. Privately owned playground equipment such as trampolines was a main cause in this group. A previous study from Das et al. also showed the risk of trampoline accidents, with traumatic brain injury and cervical spine injuries in children [23]. Further studies are needed to assess the risks and prevention options for this equipment.

The second group suffered from self-inflicted injuries or attempted suicide. These patients were predominantly female, representing 11 out of 14 patients. A similar group of attempted suicides was found by Chung et al. [22]. Prevention for this group will be mainly in the form of psychological support in the hope of preventing suicide attempts.

The cervical level was the most affected throughout all age groups, but was a relatively larger factor in younger age groups. However, the group of 0–11-year-olds was very small in this cohort. Previous studies also showed predominantly cervical spinal injuries in affected children [12]. Patients aged 12–17 years more frequently had multiple fractures, as well as fractures in multiple anatomic regions, which indicates that more extended examination is required in children where a spinal injury is diagnosed.

Spinal fractures were more common (91.2%) in 12–17-year-old patients. In the younger children 0–5 years old, we found 71.4%, and in 6–11-year-olds, we found 43.8%. Considering the physiological changes during childhood, we expected more non-osseous injuries in younger children. In the youngest group, there were still 71.4% with fractures, possibly due to confounding with a small group, or indicating that there was very high impact trauma. However, the group was too small to draw definitive conclusions.

Permanent neurological deficits were present in 14.0% of the cohort, despite the 24/7 availability of all neurosurgical and orthopedic interventions in our hospital. Considering the impact on further life for these patients, further research is needed to determine whether there are other options to restore neurological function. Other treatment strategies such as those recommended by the NASCIS study, i.e., high-dose methylprednisone in spinal trauma, were not used in this pediatric cohort [24]. Due to the lack of evidence in the pediatric group, a recent DELPHI consensus advises using steroids only for intradural surgery and not for pediatric trauma patients [25]. Further prospective studies for the prevention and treatment of neurological deficits are necessary for pediatric patients [25].

In this cohort, there was great variation in the surgical procedures and clinical neurological deficits. Considering that each of the 24 surgical patients required a unique treatment strategy, we did not further explore this for this study.

Only two patients experienced progressive neurological deficit after trauma despite immobilization. Both patients were immobilized according to the previous ambulance protocol with a spinal board and cervical collar. These two patients received urgent surgical decompression to prevent further deterioration. In the other 112 patients, there was no documented change in neurological deficit after the initial injury.

In the Netherlands, the cervical collar and spinal board were abandoned from the ambulance protocol in 2016. This study seems to confirm that there are no signs of secondary spinal column trauma during transport. However, this is based on a small group by retrospective review of hospital charts. Patients are now immobilized on the ambulance stretcher without a cervical collar, and if they are conscious, they are instructed not to move their head. Younger children are sometimes immobilized in a vacuum splint to ease the transport. The question remains whether this is the best method of transport or whether it is safe to transport young children on their parents’ lap, which is more comfortable for both child and parent.

## 5. Strengths and Limitations

This study provides a good overview of the pediatric spinal trauma population in a level 1 trauma center. 

The retrospective nature of the study is a major limitation of the study. However, there were almost no lost data in the follow up. Due to the nature of the Erasmus Medical Center, this cohort is not representative of teaching hospitals or smaller hospitals. Furthermore, the subgroups of 0–5-year-olds and 6–11-year-olds were small despite the 12-year time frame of inclusion.

## 6. Conclusions

This study shows that the incidence of spinal trauma is high in the 12–17-year-old pediatric trauma group, at 10.0%.

However, the younger groups’ incidence is much lower, and the use of ionizing radiation is relatively high. Until there are adequate clinical predictors to use a “wait and see” policy, MRI should be used as the next step after conventional X-ray to diagnose or exclude spinal injury, especially in children 0–11 years old.

## Figures and Tables

**Table 1:** Children presenting to ED with trauma between 2010 and 2021. SI: Spinal injury, ISS: Injury Severity Score, AIS: Abbreviated Injury Score.

Age Group	Total Trauma 2010–2021 N (%)	Sex (Nmale(%))	Total: ISS AIS98 (Mean (SD))	Total: Mortality within 30 Days (N(%))	With SI N(%)	With SI: Sex (Nmale(%))	With SI: Age (Mean(SD))	With SI: ISS AIS98 (Mean (SD))	With SI: Mortality within 30 Days (N(%)) [Age Range]
0 to 5	1598 (49.8%)	938 (58.7%)	6 (7)	22 (1.4%)	7 (0.4%)	6 (85.7%)	3.41 (2.04)	22 (19)	1 (14.3%) [0–1]
6 to 11	697 (21.7%)	427 (61.3%)	8 (8)	13 (1.9%)	16 (2.3%)	9 (56.3%)	9.80 (1.65)	18 (16)	2 (12.5%) [10,11]
12 to 17	914 (28.5%)	606 (66.3%)	12 (10)	31 (3.4%)	91 (10.0%)	51 (56.0%)	15.91 (1.68)	23 (14)	6 (6.6%) [15,16,17]
All	3209	1971 (61.4%)	8 (9)	66 (2.1%)	114 (3.6%)	66 (57.9%)	14.29 (3.89)	23 (14)	9 (7.9%) [0–17]

**Table 2:** Cause of trauma in children with spinal injury.

Age Group	Violence (N(%) [% of Total Injured])	Traffic (N(%) [% of Total Injured])	Workplace Accident (N(%) [% of Total Injured])	Accident at Home (N(%) [% of Total Injured])	Sports (N(%) [% of Total Injured])	Attempted Suicide (N(%) [% of Total Injured])
0 to 5 (3)	0 (0.0%) [0.0%]	1 (33.3%) [0.68%]	0 (0.0%) [0.0%]	2 (66.6%) [0.18%]	0 (0.0%) [0.0%]	0 (0.0%) [0.0%]
6 to 11 (14)	0 (0.0%) [0.0%]	6 (42.9%) [3.7%]	0 (0.0%) [0.0%]	6 (42.9%) [1.9%]	2 (14.2%) [2.7%]	0 (0.0%) [0.0%]
12 to 17 (77)	2 (2.6%) [3.1%]	35 (45.5%) [10.9%]	2 (2.6%) [10.0%]	18 (23.4%) [12.0%]	6 (7.8%) [4.7%]	14 (18.2%) [51.9%]
Total (94)	2 (2.1%) [2.2%]	42 (44.7%) [6.7%]	2 (2.1%) [10.0%]	26 (27.7%) [1.6%]	8 (8.5%) [3.8%]	14 (14.9%) [51.9%]

**Table 3:** Levels of injury.

Level of Injury	Total	0–5 Years	6–11 Years	11–17 Years	Contusion	Single Vertebra (%)	Multiple Vertebrae (%)	Multiple Regions (%)	Traumatic Brain Injury (%)	ISS AIS98 (Mean (SD))
Cervical	54	3	12	37	4	21 (38.9%)	13 (24.1%)	16 (29.6%)	23 (42.6%)	21 (13)
Thoracic	48	1	2	44	1	11 (22.9%)	12 (25.0%)	24 (50.0%)	23 (47.9%)	26 (14)
Lumbar	38	3	3	32	0	12 (31.6%)	9 (23.7%)	17 (44.7%)	13 (34.2%)	22 (15)
Sacral	5	0	1	4	0	1 (20.0%)	1 (20.0%)	3 (60.0%)	1 (20.0%)	20 (7)
Total (114)	-	-	-	-	5	45 (39.5%)	35 (30.7%)	29 (25.4%)	52 (45.6%)	23 (14)

## Data Availability

Due to privacy legislation, we are unavailable to provide the full data.

## Supplementary Materials

The following supporting information can be downloaded at: https://www.mdpi.com/article/10.3390/jcm13082372/s1, Addendum A.
