# Supplementary material for: Traumatic Spinal Injury in Children; Time to Revise Pre-Hospital and Diagnostic Protocols?
Source: J Clin Med. 2024 Apr 18;13(8):2372. doi: 10.3390/jcm13082372 (PMC11051567; doi:10.3390/jcm13082372)
Supplement: Supplementary file 1 [file jcm-13-02372-s001.zip › jcm-2948526-supplementary.pdf]

Supplementary Materials

Age	Level	Symptoms	Interventions	Recovery
15	Cervical	Partial sensory both hands	None	Full recovery
13	Cervical	Partial sensory and motor deficit 1 arm	Surgery and collar	Full recovery
16	Cervical	Partial sensory and motor deficit both arms	Surgical	Full recovery
15	Cervical	Partial sensory and motor deficit 1 arm	HALO vest, later surgical	Full recovery
16	Lumbar	Partial sensory deficit legs, bladder dysfunction	Surgical	Partial recovery
16	Thoracic	Complete motor and sensory	Surgical	No recovery
17	Cervical, thoracic, lumbar	Partial sensory and motor deficit	Surgical	Partial recovery
16	Cervical	Complete sensory and motor deficit legs, partial sensory and motor deficit arms	Surgical	Partial recovery upper extremity
15	Cervical	Complete sensory and motor deficit	None	Full recovery
16	Lumbar	Complete motor, partial sensory	Surgical	Partial recovery
13	Cervical	Partial motor and sensory unilateral arm/leg	HALO vest	Partial recovery
16	Lumbar	Partial motor and sensory 1 arm	None	Partial recovery
13	Sacral	Partial sensory	Surgical	Partial recovery
13	Cervical	Partial sensory and motor 1 arm	Vista collar	Full recovery
17	Thoracic	Complete sensory and motor deficit	Surgical	No recovery
8	Cervical	Partial sensory and motory deficit	Surgical	Full recovery
14	Cervical	Complete sensory and motor deficit	Surgical	No recovery
5	Thoracic	Complete sensory and motor deficit	Surgical	No recovery
9	Cervical, thoracic	Complete sensory and motor deficit	Surgical and HALO vest	Partial
16	Cervical	Clinically undetermined due to severe traumatic brain injury	Cervical collar	Undetermined
15	Thoracic	Partial sensory and motor deficit 1 leg	Surgical	Partial recovery
15	Cervical	Partial sensory and motor deficit all limbs	Surgical	Partial recovery
10	Cervical, thoracic	Partial sensory legs	None	Full recovery
14	Sacral	Partial sensory	None	Full recovery
17	Cervical	Partial sensory and motor arms	Surgical	Partial
16	Lumbar	Partial sensory legs	Surgical	Full recovery
